# Integrating multi-omics data to reveal the host-microbiota interactome in inflammatory bowel disease

**DOI:** 10.1080/19490976.2025.2476570

**Published:** 2025-03-10

**Authors:** Fengyuan Su, Meng Su, Wenting Wei, Jiayun Wu, Leyan Chen, Xiqiao Sun, Moyan Liu, Shiqiang Sun, Ren Mao, Arno R. Bourgonje, Shixian Hu

**Affiliations:** a Institute of Precision Medicine, The First Affiliated Hospital of Sun Yat-Sen University, Guangzhou, China; b Department of Gastroenterology, The First Affiliated Hospital of Sun Yat-Sen University, Guangzhou, China; c The First Clinical Medical School, Nanfang Hospital of Southern Medical University, Guangzhou, China; d Zhongshan School of Medicine, Sun Yat-Sen University, Guangzhou, China; e Amsterdam UMC location Academic Medical Center, Department of Experimental Vascular Medicine, Amsterdam, The Netherlands; f Department of Gastroenterology and Hepatology, University Medical Center Groningen, University of Groningen, Groningen, The Netherlands; g The Henry D. Janowitz Division of Gastroenterology, Department of Medicine, Icahn School of Medicine at Mount Sinai, New York, NY, USA

**Keywords:** IBD, gut microbiome, host-microbiota interactome, multi-omics, methodologies

## Abstract

Numerous studies have accelerated the knowledge expansion on the role of gut microbiota in inflammatory bowel disease (IBD). However, the precise mechanisms behind host-microbe cross-talk remain largely undefined, due to the complexity of the human intestinal ecosystem and multiple external factors. In this review, we introduce the *interactome* concept to systematically summarize how intestinal dysbiosis is involved in IBD pathogenesis in terms of microbial composition, functionality, genomic structure, transcriptional activity, and downstream proteins and metabolites. Meanwhile, this review also aims to present an updated overview of the relevant mechanisms, high-throughput multi-omics methodologies, different types of multi-omics cohort resources, and computational methods used to understand host-microbiota interactions in the context of IBD. Finally, we discuss the challenges pertaining to the integration of multi-omics data in order to reveal host-microbiota cross-talk and offer insights into relevant future research directions.

## Introduction

IBD is a chronic immune-mediated inflammatory disease of the gastrointestinal tract, including Crohn’s disease (CD) and ulcerative colitis (UC), and involves multiple extra-intestinal manifestations.^1^ Given the complexity, unpredictability, and heterogeneous nature of the disease, many patients suffer from therapeutic failure. More specifically, up to 30% of patients fail to initial treatment, and the rate of response lost during follow-up therapy reaches 50%, highlighting the need for a better understanding of IBD pathogenesis.^2^

Alterations in the gut microbial composition have been considered as a primary characteristic of IBD,^3^ manifesting as reduced microbial diversity and richness, along with an expansion of potentially pathogenic microorganisms. This compositional change leads to microbial functional abnormalities, including altered bacterial genome elements, transcriptional products and secreted molecules, which subsequently affect host immunological and metabolic homeostasis. Moreover, the cross-talk between microbiota and the host is complicated by personal factors, for instance, the distinct host genetic architecture, patients’ clinical history, and environmental exposures.^1^ It should be noted that many risk factors of IBD have been investigated independently by numerous studies; however, efforts to integrate the massive information generated by these single layers of biological data are lagging behind. Therefore, it is necessary to combine multiple layers of data for better characterization of disease susceptibility.

As multiple pathogenic factors are believed to trigger IBD, studying host-microbiota cross-talk through integrating different interacting components, referring to a concept *interactome*, has proven value as a systematic strategy to reveal the key drivers of disease.^4^ The concept of the interactome encompasses the integration of various layers of multi-omics-derived biological data, including both host molecules as can be profiled by the metatranscriptome, metaproteome and metabolome, and high-dimensional data deciphering the microbial metagenome. In addition to generating high-resolution biological data, the interactome also integrates the impact of internal (e.g., host genetics, immune response) and external (e.g., diet, antibiotic use, lifestyle) factors,^5,6^ which make modeling of host-microbiota interactions more precise. Moreover, the accelerated emergence of new technologies and bioinformatic methods has allow to shed light on the “dark matter” - comprising previously unknown biological structures and networks. These advancements have the potential to significantly contribute to advances in the diagnosis, prognosis and development of novel therapeutic targets.

In this review, we present an overview of how the gut microbiota interacts with the host from a multi-omics perspective, illustrating up-to-date research progress and relevant biological mechanisms. Subsequently, we summarize multi-omics technologies, bioinformatic methodologies and cohort resources facilitating interactome studies. Finally, we outline several key challenges and future perspectives in the field.

## Host-microbiota interactome in IBD

### Microbial composition

Dysbiosis has been widely observed, not only in IBD case-control studies, but also across several disease subtypes.^7^ Compared to healthy individuals, it has been reported that there is a significant decrease in microbial abundance and diversity of commensal bacteria (e.g., Firmicutes and *Bacteroides*),^8–10^ with an enrichment of Proteobacteria (e.g. adherent-invasive *Escherichia coli* (AIEC))^11^ in patients with IBD. At species level, although disease-associated taxa vary across studies, some core species, including *Roseburia intestinalis, Faecalibacterium prausnitzii*, and *Akkermansia muciniphila*, consistently show decreased abundances during both disease activity and remission stages, representing long-term gut microbial dysregulations in IBD.^12,13^ Additionally, certain microbial changes can be specific to subphenotypes of IBD. For example, a multi-regional study has revealed that CD and UC possess distinct microbial communities based on fecal samples.^14^ One study, using paired inflamed and non-inflamed mucosal biopsies, proved higher colonization ability of *Cloacibacterium* and *Tissierellaceae* in disease sites.^15^ Besides, changes in the microbiome can also indicate various disease states. Libertucci et al. identified tissue bacteria as a strong indicator of mucosal healing.^16^ Neut *et al*. showed that patients who underwent ileocecectomy were more likely to develop postoperative recurrence of CD when there were elevated levels of *E. coli* and *Bacteroides*.^17^ Patients with CD who with low levels of *F. prausnitzii* in the mucosa tended to experience relapse after surgery.^18^ Conversely, the reestablishment of *F. prausnitzii* after recurrence was linked to sustained clinical remission in UC.^19^

In addition, in patients with IBD, fungal dysbiosis has also been observed, manifested by an increased *Basidiomycota*-to-*Ascomycota* ratio, a diminished relative abundance of *Saccharomyces cerevisiae*, and an enhanced proportion of *Candida albicans*, compared to healthy individuals.^20^ A recent study demonstrated a greater prevalence of enteric infections (such as norovirus) as well as *Enteroinvasive Escherichia coli* (EIEC) in both CD and UC patients during disease exacerbations.^21,22^ Accumulating evidence also suggests viral involvement in the interactions between bacteria and yeast species.^23^ Compared to healthy individuals, the abundance of *Methanosphaera stadtmanae* was increased 3-fold in patients with IBD;^24^ the prevalence of *Methanobrevibacter smithii* was significantly reduced in IBD, but they returned to normal during disease remission.^25^ However, compared to intestinal bacteria, the precise function of gut fungal and viral populations associated with IBD remains largely undefined, necessitating more focus in future studies.^26,27^ Collectively, these studies highlight the role of the gut microbiome in IBD disease progression. In treatment, the use of small molecule biological agents, probiotics, and fecal microbiota transplantation (FMT) may significantly alter the gut microbiome. Some difficulties have been raised when restoring gut microbial composition in humans during FMT. The transplant efficiency is dramatically influenced by the selection of donors, microbiota-intestine compatibility in recipients and the colonization capability of transplanted microorganisms.^28–30^ Thus, a deeper exploration beyond microbial composition is required to fully capture the nature of intestinal dysbiosis in IBD.

### Microbial genetic variation

Investigation of the microbial genomic architecture has largely extended our knowledge of the host-microbiota interactome. Compared to humans, microorganisms’ genomes are much smaller, but characterized by a complex genetic structure, including single nucleotide variants (SNV), structure variants (SV) and copy number variations (CNV). These genomic alterations lead to strain differentiation which leads to variations in microbial fitness, carbohydrate utilization, metabolizing capacity, pathogenicity and other biological properties.^31^ For example, a recent study identified a deletion of a DNA segment containing GalNAc utilization gene clusters in a group of *F. prausnitzii* bacteria. These saccharide-enzyme deficient bacteria might show lower competitive ability for intestinal mucus degradation and thus affect host’s cardiometabolic health.^32^ In patients with IBD, an increased copy number of a gene (*K08217*), encoding for a major drug efflux protein identified from *Roseburia inulinivorans*, was highly enriched by enhancing bacterial antibiotic resistance. Similarly, *HlyD* (*K01993*) harbored by *Bacteroides uniformis*, an essential component of RTX hemolytic toxin secretion, exhibited an increased copy number in IBD.^33^ Collectively, these studies have indicated that analyzing the genetic architecture of the gut microbiota may help to unveil novel pathogenetic mechanisms in IBD, and they could provide a more profound understanding of microbial evolution and adaptation within the human intestinal ecosystem.

The characteristics of microbial genetic architecture, unlike abundance, have also shown potential diagnostic utility for IBD. While a decreased abundance of *F. prausnitzii* has been demonstrated in patients with a variety of immune-mediated diseases, not only IBD but also type 2 diabetes (T2D), colorectal cancer (CRC), and psoriasis,^34,35^ it has presented poor discriminative performance between these diseases. However, one study incorporated SNVs from *F. prausnitzii* and *Eubacterium rectale* and could accurately identify IBD from other diseases based on this information.^36^ Another study demonstrated that microbial genes outperformed taxonomic abundance in distinguishing CD from CRC, T2D, Parkinson’s disease (PD), and liver cirrhosis (LC).^37^ Notably, a recent large-scale study discovered the microbial genetic make-up was strongly associated with geographical factors,^38^ suggesting that the specificity of genetic markers used for diagnosis, should be carefully reevaluated in much larger and diverse populations.

### Microbial gene expression

Although microbial functionality can be inferred from their genome, measuring the transcriptome allows the quantification of which genes are actually expressed, thereby allowing to characterize the potential functional activity of the microbiota.^39^ An early study defined a cluster of “dormant” intestinal microbiota which was highly enriched but with inactive functions in patients with IBD, by comparing bacterial gene copies and transcription levels. That was the first time to reveal the differences in the qualitative composition between DNA numbers and gene expressions.^40^ This observation has been further confirmed by a later study using metatranscriptome sequencing with a much larger case-control design. One prominent example was that the RNA amplification of *Ruminococcus gnavus* was three times that of its DNA abundance, indicating small changes in DNA level of certain bacteria could be more functionally impactful. Another example is that strong transcriptional activity of isoprenoid production was examined in *Alistipes putredinis* in patients with severe IBD, but without a notable increase of relevant gene copies.^41^ Thus, disturbances associated with disease that are not identified at the microbial composition level may be more evident in terms of transcriptional activity.

Intriguingly, combining microbial DNA and RNA measurements has successfully mirrored IBD pathology in real-time compared with that using genome information alone.^42^ Ilott *et al*. identified that in patients suffering from active colitis, several bacteria displayed consistent patterns between genomic and transcriptomic levels for a particular group of genes. These gene families were involved in nutrient deprivation responses, antimicrobial peptide production, and oxidative stress responses, which could be used as reliable microbial markers to monitor disease activity.^43^ These findings demonstrated that intestinal bacteria might respond to environmental stressors in the host by rapidly altering transcriptional activity, with or without detectable changes in the composition of the gut microbial community, thereby reflecting host’s immune status.^44^

Transcriptome analysis offers a snapshot of gene expression, providing real-time insights into genes that are actively transcribed in a particular sample under specific conditions. This technique allows for the identification of differentially expressed genes that may be associated with disease states.^39^ However, its limitation lies in its transient nature, as it only reflects the current expression levels and does not account for long-term genomic changes or regulatory mechanisms that might affect gene function over time. On the other hand, genomic analysis offers a more stable and comprehensive view by capturing the entire genetic makeup of an individual, including both coding and non-coding regions. It can identify genetic variants that might predispose individuals to IBD or other diseases, regardless of their current expression. However, genomic analysis alone cannot directly capture dynamic gene activity, and the interpretation of genetic variants often requires further functional validation.

### Microbial protein functionality

Protein determination complements microbial functionality profiled by transcriptional information since proteins are the basis of membrane structures, signaling messengers and secreted functional units. These molecules have been recognized as important ligands for host receptors. For example, the S-layer protein A on the surface of *Lactobacillus acidophilus* NCFM acts as a ligand for DC-SIGN (dendritic cell-specific ICAM-3-grabbing nonintegrin), thereby promoting intestinal immune homeostasis by regulating dendritic cells. Activated DC-SIGN elevated the expression of downstream anti-inflammatory cytokine interleukin 10 (IL-10).^45^ In contrast, bacterial flagellin-induced host inflammation by binding to human Toll-like receptor 5 (TLR5), which upregulated the NF-κB transcription factor, leading to higher expression of various pro-inflammatory cytokines.^46^ Interestingly, recent studies have identified that bacterial genetic variants encoding flagellin proteins may lead to “silent recognition” without eliciting host inflammatory responses, and even escape from TLR5 activation.^47,48^ These findings may help to explain how the host intestinal immune system tolerates commensal flagellin, while it still remains responsive to various pathogenic flagellins. Furthermore, microbial secretory proteins also act as mediators of interactions between microorganisms and hosts. For instance, *Bacteroides vulgatus* proteases could cause colonic epithelial damage and contribute to UC disease activity.^49^ The epithelial cells treated with proteases exhibited severe damage. Another study showed that some proteases secreted by gut commensals participate in the degradation of multiple extracellular matrix (ECM) proteins, including collagen, laminin and fibronectin, which may contribute to ECM remodeling and affect intestinal fibrosis.^50^ Taken together, microbial proteins influence host immunity and mucosal homeostasis as biofilm components or in secreted form. Exploration of microbial proteins has unraveled host-microbiota protein–protein networks^51^ and provided novel therapeutic opportunities.

### Host and microbial metabolites

While proteins are the direct products of the genome, metabolites have been considered as small molecules resulting from the interaction between host, microbiota and other exposome factors, such as diet and medication. Several classes of metabolites are differentially abundant in patients with IBD compared with healthy individuals, including short chain fatty acids (SCFAs), sphingolipids, bile acids, amino acids and their derivatives.^52^

#### Short-chain fatty acids (SCFAs)

SCFAs are produced by microbial fermentation of dietary fiber and exert multiple effects on host metabolism and the immune system. It has been consistently reported that patients with IBD typically exhibit a decrease in fiber-fermenting and SCFA-producing bacteria compared with healthy controls.^53^ A recent study demonstrated the decreased SFCA-production by *Faecalibacterium, Dorea*, and *Fusicatenibacter* might contribute to disease relapse instead of being the consequence of inflammation, through a healthy first-degree relative (HFDRs) family-based cohort design.^54^ SCFAs constitute as carbon source for the intestinal epithelium and many kinds of bacteria. For example, *Akkermansia muciniphila* utilizes the carbohydrates found in mucus as an energy source, breaking them down into oligosaccharides and acetate within the intestinal microenvironment, which in turn nourish other bacterial species.^55^ These molecules are subsequently taken up by bacteria like *Eubacterium hallii*, which can then synthesize propionate, butyrate, and vitamin B12. These metabolites are released into the lumen, where they exert beneficial effects on colonocytes.^56^ Furthermore, SCFAs also function as ligands for G-protein coupled receptors (GPCRs). For example, GPR43 mediates the inflammatory regulatory effects by recruiting neutrophils in a “acetate-sensing” manner.^57^ Another example is butyrate, which may mitigate the loss of mitochondrial membrane potential induced by *E. coli-LF82* through FFAR3/GPR41 receptor-mediated pathways, exerting a protective effect against mucosal damage.^58^ Another study demonstrated that butyrate can alter the phenotype of IL-4-induced macrophages (M(IL-4)s) by inhibiting histone deacetylation, enhancing their phagocytic capacity.^59^ Animal experiments further reveal the role of SCFAs in the gut. In mouse models of colitis, SCFAs have been demonstrated to elicit the production of IL-10 in Th1 cells and upregulate the expression of antimicrobial peptides (AMPs) in intestinal epithelial cells, via the activation of signal transducer and activator of transcription 3 (STAT3) and the mammalian target of rapamycin (mTOR) signaling pathways.^60,61^ Additionally, SCFAs have been shown to ameliorate intestinal inflammation by modulating components of innate immunity through the inhibition of Toll-like receptor 4 (TLR4) expression and the differentiation of Th17 cells in chemically induced colitis.^53^ SCFAs play a role in the maintenance of intestinal barrier integrity and immune regulation by the aforementioned pathways.

#### Sphingolipids

All eukaryotic membranes contain sphingolipids (SLs), a group of lipids consisting of a sphingosine backbone and a fatty acid. Microbiota can release sphingosine or other metabolic products through both self-degradation and the metabolism of the human sphingolipids, including ceramide, sphingosine-1-phosphate (S1P), and ceramide-1-phosphate, which are important molecules promoting epithelial integrity and mucosal inflammation. For example, the SphK1-S1P-S1PR1 axis has a significant role in IBD, with increased S1P levels and elevated SphK1 expression observed during colitis. S1P influences mucosal barrier function, as evidenced by its ability to elevate the level of E-cadherin, a crucial adherens junction protein, and thereby enhancing the integrity of the barrier.^62^ S1P receptor modulators (e.g., ozanimod), which have recently been added to the therapeutic arsenal of IBD, also bind to the S1P receptors and interact with S1P1 to regulates lymphocyte egress from the spleen and lymph nodes into the systemic circulation.^63^ Reduced lymphocyte egress leads to a lower number of circulating lymphocytes in the bloodstream, which subsequently results in decreased inflammation and less tissue damage.^64^ Increased activity of SphK1 has also been shown to modulate macrophage recruitment and strengthen their anti-inflammatory properties.^65^ One of the constituents of sphingolipids, sphingosine, also shows anti-inflammatory activity. Sphingosine can inhibit LPS-induced activation of NF-κB signaling pathway, repressing the production of inflammatory cytokines.^66^ Ceramides play a crucial role as membrane lipids and also function as second messengers in cellular signaling pathways. Six distinct human CerS enzymes have been characterized, each generating ceramides with varying chain lengths based on substrate specificity.^67^ For example, a previous study demonstrated that CerS6 deficiency exacerbated inflammation in a mouse model of dextran sulfate sodium (DSS)-induced colitis.^68^

#### Bile acids and their derivatives

Bile acids (BAs) are endogenous metabolites produced by both intestinal microbiota and the host. The intestinal microbiota participates in various biotransformation reactions on host-derived primary BAs to produce secondary BAs. For example, deconjugation was mainly carried out by microbial bile salt hydrolases (BSH) gene cluster, which prevented primary BAs reabsorbed from the intestine and enables follow-up dehydroxylation. The secondary BAs exerted various metabolic and immune effects by binding to receptors such as transmembrane G protein-coupled receptor 5 (TGR5), farnesoid X receptor (FXR), vitamin D receptor, pregnane X receptor, and constitutive androstane receptor.^69^

In patients with active IBD, fecal levels of conjugated (primary) BAs are significantly elevated, while levels of secondary BAs are markedly decreased.^70^ Secondary BAs exhibit higher affinity for TGR5 than primary BAs.^71^ Activation of the TGR5 receptor can modulate the phenotypic conversion of macrophages toward an M2-predominant anti-inflammatory phenotype, inhibiting the production of NF-κB-related pro-inflammatory cytokines.^52^ Therefore, the decrease in secondary BA levels may contribute to IBD susceptibility by affecting the anti-inflammatory pathway of TGR5. Another study demonstrated that BAs play a crucial role in the development of RORγ^+^ regulatory T cells (Tregs) in the colonic mucosa or lamina propria. The number of Tregs could be significantly reducted by eliminating the BA metabolic pathway in mice intestinal bacteria.^72^ Devkota *et al*. found that a high-fat dairy diet increases the proportion of taurine-conjugated bile acids in mice, leading to a proliferation of *Wadsworthia* which metabolizes sulfur from taurine to toxic hydrogen sulfide,^73^ indicating a bidirectional effect between BAs and gut microbiota.^74^

#### Amino acids

The production of amino acids by the gut microbiota, which shows many alterations in patients with IBD, is crucial to intestinal homeostasis. An ubiquitous negative correlation has been identified between serum tryptophan (Trp) and the systemic inflammatory marker C-reactive protein (CRP).^75^ Xanthurenic acid (XANA) and kynurenic acid (KYNA), metabolites of tryptophan, can reduce the severity of colitis by influencing intestinal epithelial cells and T cells, through the activation of the aryl hydrocarbon receptor (AhR) and the reconnection of cellular energy metabolism. Furthermore, the direct regulation of endogenous tryptophan metabolism using recombinant aminoadipate aminotransferase (AADAT) to generate XANA and KYNA shows protective effects in mice models.^76^ These studies suggested that supplementation of tryptophan and its derivates holds potential application value in the treatment of IBD. Arginase 1 (Arg1), which catalyzes the conversion of L-arginine into ornithine and urea, exhibits pleiotropic immunoregulatory effects. Studies have shown that the expression of Arg1 correlated with the extent of inflammation in the intestinal tissues of patients with IBD. While an L-arginine-free diet attenuated the pro-resolving effects of Arg1 deficiency, concurrent deletion of other L-arginine-metabolizing enzymes, such as Arg2 or Nos2, restores its protective effects, highlighting the critical role of L-arginine in alleviating colitis.^77^

**Figure 1.**
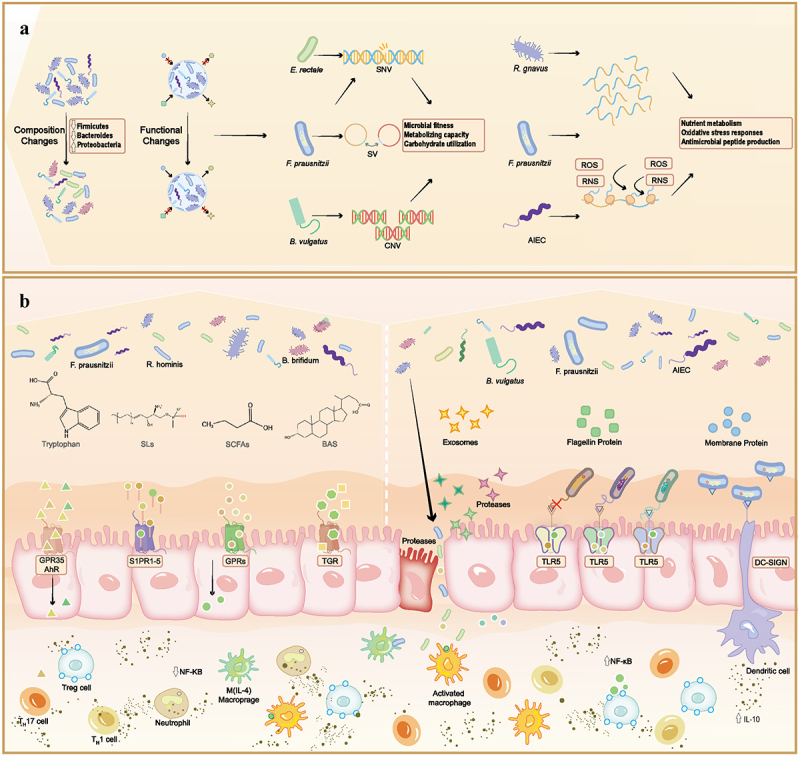
Host-microbiota interactome in IBD. (a) Compositional and functional characteristics of the gut microbiota are both altered in IBD. Changes in the composition are reflected in a significant decrease in microbial abundance and diversity of certain commensal bacteria (e.g., Firmicutes and *Bacteroides*), with an enrichment in potentially pathogenic bacteria such as Proteobacteria. Functional changes, on the other hand, are reflected in alterations at different omics levels. The bacterial genome can significantly affect the host through variations in SNV, CNV, and SV. At the transcriptomic level, RNA can influence host’s nutrient metabolism, oxidative stress responses, and antimicrobial peptide production. (b) Proteins and metabolites can directly interact with intestinal epithelial cells. For example, membrane proteins can directly bind to the DC cell receptor DS-SIGN, leading to an increase in IL-10 secretion and a reduction in inflammation. Flagellar proteins can interact with the TLR5 receptor, activating the Nf-κB signaling pathway. Additionally, secreted proteins from *B. vulgatus* can damage intestinal epithelial cells. Small-molecule metabolites (e.g. SCFAs, SLs, BAs, TRPs) can act on receptors on epithelial cells, thereby influencing T cells (T regulatory cell, T_H_17 cell, T_H_1 cell) and macrophages to regulate the inflammatory state. Abbreviations: *E. rectale*: *Eubacterium; F. prausnitzii*, *faecalibacterium prausnitzii; B. vulgatus*, *Bacteroides vulgatus; R. gnavus*, *ruminococcus gnavus; R. hominis*, *Roseburia hominis; B. brifidum*, *Bifidobacterium brifidum; AIEC, Adherent invasive E coli;* SCFAs, short-chain fatty acids; SLs,Sphingolipids; BAs, bile acids; GPCR, G-protein coupled receptor; AhR, Aryl hydrocarbon receptor; S1PR, Sphingosine-1-phosphate receptor; TGR5, Transmembrane G protein-coupled receptor 5; TLR5, toll-like receptor 5; Nf-κB, nuclear factor-κB.

The differences in metabolic profiles between healthy individuals and patients with IBD have rendered quantitative analyses of a vast array of molecules for developing disease biomarkers.^78^ However, most current studies have focused on either blood or stool, where disparate patterns were discovered between these two biological samples.^79^ Considering tissue-wide metabolomic investigation in further studies may reveal a more disease-relevant spectrum.^80,81^ (Figure 1 illustrates the host-microbiota interactome.)

### Other factors

Host and environmental factors also play an important role in shaping host-microbiota interactions. The composition of the gut microbiota differs among individuals and changes over time during development. This variability is influenced by the host’s genotype as well as various environmental factors. Specifically, dietary habits and antibiotic usage have been shown to play significant roles in the establishment and maintenance of microbial diversity within the gut.^82^ A Mendelian randomization study revealed an enrichment of microbiome-related genomic loci within the metabolic, nutritional, and environmental domains.^83^ Another study reported a strong increase of Bifidobacterium levels in genetically lactose-intolerant people,^84^ suggesting that host genetic make-up may shape the gut microbiota. Higher consumption of animal-derived, processed foods, alcohol, and sugar links to a pro-inflammatory microbial environment and elevated inflammatory markers. In contrast, plant-based diets are associated with the presence of SCFA-producing bacteria, microbial metabolism of polysaccharides, and a reduced abundance of potentially harmful bacteria.^85^ Llewellyn et al. have shown that dietary protein can exert a detrimental effect on colitis development.^86^ This phenomenon may be attributed to altered gut microbiota resulting from a diet low in fibers, which could deteriorate the mucus layer, and increase infection susceptibility and chronic inflammation.^87^ Another study indicated that a maternal high-fiber diet during pregnancy and lactation modifies the thymic microenvironment, upregulating T-cell maturation. Enhanced fiber intake has also been shown to raise blood butyrate levels in offspring, as well as GPR41-dependent boosting of Treg cell counts in both the periphery and thymus.^88^ On the other hand, antibiotic disturbances can lead to reduced overall microbial diversity and a higher abundance of facultative anaerobes, such as E. coli and Salmonella spp.^89,90^

## Multi-omics technologies

**Figure 2.**
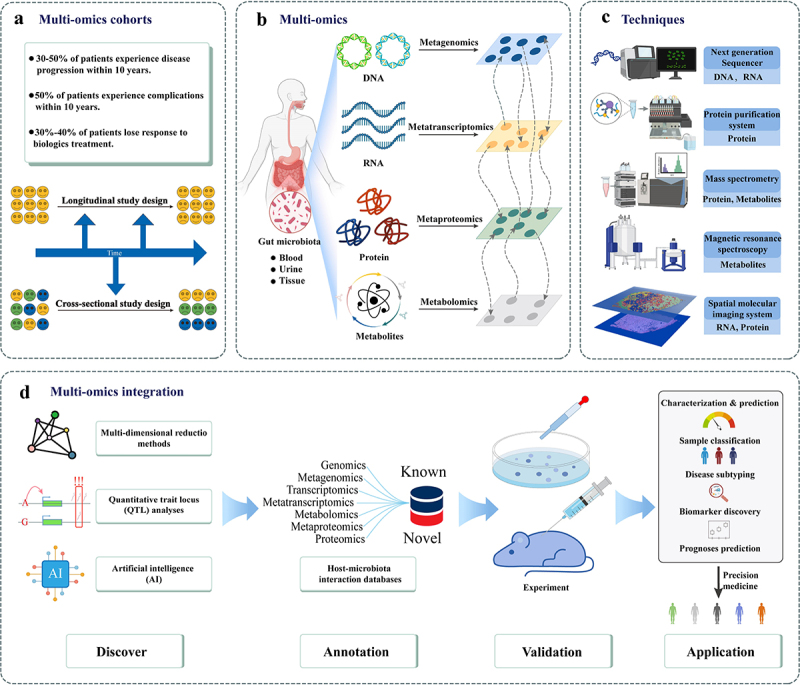
Overview of multi-omics integration to reveal host-microbiota interactome. (a) The design of different cohorts to address various clinical issues in IBD. (b) Detection of host blood, urine, or tissue samples to analyze DNA, RNA, proteins, and metabolites of the gut microbiota. (c) Using high-throughput detection technologies to analyze different molecules. (d) Various multi-omics integration methodologies support multi-omics studies.

The advances in high-throughput technologies has boosted the generation of multi-omics data and facilitated the discovery of unknown molecules. In the subsequent section, we summarize (Table 1) key multi-omics technologies that are characterized by high precision, high dimensionality, and the ability for high-throughput analysis (Figure 2).

**Table 1.** Overview of key multi-omics technologies including a brief description and examples of platforms commonly used for each technology.

Omics	Description	Example of technological platforms used
Metagenomics	Culture-independent analysis of genomic sequences from all microbes in a sample; reveals information on the taxonomic and functional profiles of microbial communities.	Ion Torrent Semiconductor Sequencing, Illumina Sequencing, Sequencing by Oligonucleotide Ligation and Detection, DNA Nanoball Sequencing, PacBio Single Molecule Real-Time (SMRT) Sequencing, Nanopore DNA Sequencing
Metatranscriptomics	Culture-independent analysis of microbial community transcriptomes; discerns active microbes and their functional expressions under specific conditions.	
Proteomics	Systematic analysis of the proteome; elucidates protein interactions, functions, and structures to provide deeper insights into cellular activities and organismal function beyond genomics.	Mass Spectrometry, Antibody Capture-based Techniques, X-ray Crystallography, Nuclear Magnetic Resonance Spectroscopy, Liquid Chromatography
Metabolomics	Systematic quantification and characterization of small-molecule metabolites in biological samples, reflecting the metabolic state and functional outputs of cellular processes.	Mass Spectrometry, Nuclear Magnetic Resonance Spectroscopy, Liquid Chromatography, Gas Chromatography, Capillary Electrophoresis
Spatial transcriptomics	Omics technique building upon in situ hybridization; captures the spatial context of transcriptional activity within intact tissues and quantifies all mRNA in cells to provide a comprehensive view of cellular processes and their spatial organization.	Multiplexed fluorescence in situ hybridization (M-FISH), Single-cell RNA Sequencing
Spatial proteomics	High-resolution analytical approach that integrates proteomic profiling with spatial localization, elucidating protein distribution, dynamics, and interactions within cellular and tissue contexts.	Cyclic Immunofluorescence (CycIF), Co-detection by Indexing (CODEX), Iterative Bleaching Extends Multiplexity (IBEX), Imaging Mass Cytometry (IMC), Multiplexed Ion Beam Imaging (MIBI), Antibody-Based Imaging, Fluorescent Protein-Based Imaging

### High-throughput sequencing (HTS)

HTS has revolutionized the profiling of nucleotide-unit based molecular traits,^91^ from the first-generation sequencing technology, exemplified by Sanger sequencing,^92^ to the next- and third-generation sequencing technologies which have dramatically increased sequencing lengths and efficiency. This technical evolution helped to consider the host-microbiota interactome as an ecosystem and explore the multi-layered communication in terms of genome, transcriptome and epigenome. Currently, shotgun metagenomic sequencing is a culture-independent approach that involves all microorganisms from the environment, showing clear advantages over 16S rRNA sequencing to cover the entire microbial genome and explore potentially unknown species.^93,94^ For instance, multi-kingdom integration of bacteria, archaea and fungi has presented a better accuracy for disease diagnosis^95^ and prediction of therapeutic response.^96^ Community-level analysis, referring to enterotypes or microbial networks, also reflects the role of ‘core taxa’, which are defined as a group of bacteria that are dysregulated across different diseases.^97^ To better characterize the complex genomic or transcriptional structures, third-generation sequencing examines long-read DNA molecules without breaking them into small pieces. Several recent studies have recovered the bacterial genome with accurately identified SNVs and SVs based on long-read sequencing methods.^98,99^ Moreover, although still in its infancy, microbial single-cell sequencing is an emerging technology that helps to capture the functional dynamics of single bacteria.^100^ Genomic sequencing provides a stable and comprehensive insight into genetic variations, helping to identify genetic predispositions and potential therapeutic targets in IBD. In contrast, transcriptomic sequencing quantifies gene expression changes that reflect the dynamic immune and inflammatory processes in IBD, making both methods complementary for understanding disease mechanisms.

### High-throughput proteomic platforms

The high-throughput measurement of proteins (proteomics) is accompanied by several technical difficulties. First, while an organism’s genome is relatively stable, proteins exhibit considerable fluctutations across different cell types and time points. Second, the maturation of proteins involves diverse chemical modifications (such as phosphorylation and ubiquitination). This implies that protein identification needs to consider molecular diversity and spatial structure.

Mass spectrometry (MS) and antibody capture-based techniques are two commonly used methods. MS can be combined with various separation and pre-fractionation methods (e.g. liquid chromatography, LC) to precisely identify the desired protein or peptide, thereby improving identification accuracy and output.^101,102^ Compared to MS, antibody-based and aptamer-based techniques demonstrate superior efficacy in terms of sensitivity and detection throughput. For example, Olink and SOMAscan assays can simultaneously detect thousands of proteins using a minuscule amount of sample (e.g., a few microliters). However, it is difficult to directly compare the different analytical aspects since only ~1,000 highly present proteins can be detected in multiple approaches.^103^ When choosing proteomic methods, researchers should carefully consider their study purpose, detection panels, and technical costs.

Recently, X-ray crystallography and Nuclear Magnetic Resonance (NMR) spectroscopy techniques have been applied as structural proteomics techniques. This enables the comparison of protein structures and assists in identifying the functions of newly discovered molecules.^104^ Structural information also aids in understanding where drugs bind to proteins and reveals interactions between proteins. For instance, when combining NMR spectra with machine learning algorithms, it was possible to identify the three-dimensional structures of over a hundred proteins within a few hours, enabling to study their interactions. This can also be effectively used to investigate drug-protein interactions by structurally analyzing drug targets in cells or tissues.^105^ Given the complex nature of IBD and the need for precise understanding of protein functions and interactions, these techniques hold significant potential for uncovering disease mechanisms and discovering new therapeutic targets in IBD in the future.

### High-throughput metabolomics

Metabolism represents the ultimate product of cellular reactions. Quantifying thousands of small molecules may serve as a functional readout of host-microbiota physiological states.

NMR spectroscopy-based metabolomics is employed for untargeted identification and semi-quantification of metabolites. The intensity of the ^1^H NMR spectrum directly correlates with the proton count in a molecule, enabling high-resolution NMR spectroscopy to quantify metabolites in an untargeted manner. Proton resonance in an NMR spectrum reflects the chemical environment, providing crucial common molecular structural information for metabolite identification.^106^ MS is often coupled with separation techniques such as LC, gas chromatography (GC), and capillary electrophoresis (CE). MS-based metabolomics can be categorized into targeted and untargeted approaches. The targeted approach selectively quantifies specific metabolites, a class of metabolites, or metabolites in a pathway, such as BAs or amino-containing metabolites, by incorporating of internal standards.^107,108^ Untargeted approaches have the advantage to explore unknown metabolites which can typically generate approximately 10,000 features depending on the profiling method and analytical platform. It is still very challenging to perform cross-cohort and validation studies using different technologies, because they are not always compatible with each other and thus usually have few overlap between detected metabolites. Therefore, researchers should carefully consider their choice of which methodology to use that should be driven by the specific study purposes.

## IBD multi-omics cohorts

The development of high-throughput platforms has provided a unique opportunities for studying the host-microbiota interactome in large-scale patient cohorts. Unlike animal studies that generally focus on understanding causality and investigating underlying pathogenic mechanisms, multi-omics-based data generation in patient cohorts facilitates capturing the complexity of these interactions on a population level.^109^

External environmental exposures, such as dietary habits, may impact the risk of IBD by shaping the composition of the microbiome. With the development of socioeconomic standards, there has been an increased variety, processing, and formulation of foods. This has led to a higher consumption of animal-based, high-calorie, high-fat, and processed sugar diets, which are generally lower in dietary fiber,^110^ leading to reduced microbial diversity.^111^ Additionally, microbial functionality, such as metabolite pools (acylcarnitines, BAs, and SCFAs) and levels of antibodies in host is significantly associated with disease activity across a series of timepoints during the disease course.^112,113^ This suggests that host-microbiota interactions are not static, which poses a challenge to host-microbiota interactome studies. This requires the analysis of preferably longitudinal patient cohorts with well-documented phenotypic- and multi-omics data. (Table 2)

**Table 2.** Characteristics of population-level microbiome cohort studies in IBD.

Cohort name	Numer of participants	Data generated	Study features	Reference
RISK(USA)	n = 1,276	Genomics, Transcriptomics, Microbiomics; Blood, Biopsies, Faecal samples	Longitudinal, CD only, pediatric inception cohort, multi-omic analysis	114,115
PRISM(USA)	n = 161	16S rRNA sequencing, Metabolomics, Microbiomics; Faecal samples	Longitudinal,	116,117
Dutch IBD biobank(Netherlands)	n = 3,388	Genomics, Transcriptomics, Microbiomics; Serum, Faecal, Mucosal biopsies sample	Cross-sectional, multi-omic analysis	118
The Swiss Inflammatory Bowel Disease Cohort Study (SIBDCS,Switzerland)	n = 3,577	16S rRNA sequencing, mGenomics, Microbiomics; Blood, Faecal, mBiopsies samples	Epidemiology, multi-omic analysis	119
IBD BioResource (UK)	n = 36,126	Genomics; Serum plasma samples	Longitudinal	120
1000IBD(Netherlands)	n = 1,215	16S rRNA sequencing, Genomics, Stool, Biopsies samples	Cross-sectional, multi-omic analysis	121
PANTHER (Belgium)	\	Genomics; Stool, Serum, Mucosal biopsies sample	Longitudinal, Multi-center, follow-up	122
Human Microbiome Projec 2 (HMP2,USA）	n = 132	Microbiomics; Stool, Blood, Biopsy samples	Longitudinal, Multi-omic analysis	113,123
The Crohn’s and Colitis Canada Genetic Environmental Microbial project(GEM project,Canada)	n = 3,483	16S rRNA sequencing, Metabolomics, Microbiomics; Faecal samples	Family-based, prospective cohort study of healthy first-degree relatives(FDRs) of individuals with CD	124
TWIN-IBD (Netherlands)	\	Blood, urine, feces, Oropharyngeal swabs, Rectal, colonic or ileal biopsies samples	Family-based, ongoing, prospective, Longitudinal, follow-up, twins only ≥16 years of age	125
The Inflammatory Bowel Disease Family Cohort (IBD-FC, Germany)	n = 1,715	Fecal, Blood samples	Family-based, Prospective, follow-up	126
Predicting Response to Standardized Colitis Therapy (PROTECT, USA and Canada)	n = 431	16S rRNA sequencing, Metabolomics, Microbiomics; Faecal, Biopsies samples	Treatment-naive pediatric UC patients	127

IBD, inflammatory bowel disease; CD, Crohn’s disease; UC, ulcerative colitis.

### Family-based cohorts

Twins- and relative-based cohorts studies provide the opportunity to search for disease-driving factors independent of host genetics and environmental exposures. A recent study from the Dutch TWIN-IBD consortium has discovered that the gut microbiome of healthy cotwins in IBD-discordant twin pairs displayed IBD-like signatures before IBD onset,^125^ revealing key microbial players precede the disease. The Canada Genetic Environmental Microbial (GEM) project has enrolled hundreds of healthy first-degree relatives (FDRs) of patients with CD, followed for a median time of 5.4 years.^124^ A microbial risk score (MRS) consisting of *Ruminococcus, Blautia, Colidextribacter*, an uncultured genus of *Oscillospiraceae*, and *Roseburia*, has been established to predict healthy relatives developing CD within several years of follow-up. Moreover, researches have validated the anti-inflammatory effects of metabolites derived from these bacteria such as biotin (vitamin B_7_) and niacin (vitamin B_3_) in mice models, revealing disease-driving microbial factors through examining individuals with a similar environmental and genetic background^124^ Another study used HFDRs as controls to analyze the microbiota and metabolome in individuals with active (CD-A) and quiescent (CD-R) CD, minimizing the impact of genetic and environmental factors. Compared to unrelated controls, the use of HFDRs led to the identification of fewer differential microbial taxa, including *Faecalibacterium, Dorea*, and *Fusicatenibacter*, which were found to be reduced in CD-R and associated with SCFAs.^54^ The IBD Family Cohort (IBD-FC) was established in Germany to recruit IBD patients and their unaffected relatives, assessing microbiome changes around IBD onset. Species-level analysis revealed enrichment of *Enterocloster* species in CD and depletion of *Faecalibacterium* and *Blautia* species. High-risk relatives exhibited microbiome features transitional to IBD cases, suggesting a predisposed state.^126^ Overall, environmental factors play a crucial role in the pathogenesis of IBD. Family-based cohorts, through generally reflecting a similar living environment and controlling for genetic differences, offer an advantageous strategy for investigating disease etiology.

### Large-scale cross-sectional cohorts

The heterogeneity of IBD includes a variety of clinical signatures that include but are not limited to disease subtype, disease location, extra-intestinal manifestations and different responses to treatment. An example of a large-scale cross-sectional cohort includes the 1000IBD cohort in the Netherlands, which has collected data pertaining to both phenotypical records (diet and lifestyle behavior, therapeutic history and adverse drug events) and multi-omics (genomic, transcriptomics, proteomics, metabolomics and microbiome) from over 1,200 patients with IBD. By integrating clinical history, personal lifestyle and metagenomic data, one key study has shown that certain gut microbiota were able to metabolize drug compounds, resulting in different response to medication.^128^ Another study has revealed person-specific nutrients associated with clinical outcomes that were potentially mediated by pro- or anti-inflammatory gut microbial species, providing suggestive reference for diet intervention trials.^85^ Combining host genomics and fecal metagenomics data, researchers have extended the knowledge on interactions between host genetic factors and gut microbiota, pinpointing important mechanisms behind the disease risk.^129,130^ Another study, based on the FAMISHED cohort, comprehensively assessed the heterogeneity of gut microbiome across different IBD conditions and disease locations.^131^ In particular, terminal ileitis (CD-TI) showed a marked enrichment of *Faecalibacterium* compared to other CD subtypes. Conversely, colonic Crohn’s (CD-CC) and small bowel Crohn’s (CD-SB) were notably enriched for opportunistic pathogens; *Streptococcus* and *Burkholderia* were more abundant in CD-CC patients compared to all other disease locations, while *Escherichia* and *Acinetobacter* were more prevalent in CD-SB patients relative to other CD subtypes. However, no significant differences were observed at different disease locations across patients with UC. Collectively, all these cross-sectional studies have built the blueprint of associations between host features and gut microbiota, guiding the follow-up mechanistic and clinical research designs.

### Longitudinal cohorts

The longitudinal analysis of multi-omics data allows concurrent changes to be observed in the host together with microbial molecular activities over time. For example, the Integrative Human Microbiome Project (iHMP)^113^ collected longitudinal multi-omics data from 110 patients with IBD together with non-IBD controls over the course of 1 year. These high-dimensional multi-omics resource involved many timepoints (up to 24 timepoints for each individual) and different biological samples, including intestinal tissues (bulk transcriptomics and 16S rRNA sequencing), stool samples (metagenomics, metatranscriptomics, proteomics, and metabolomics), and peripheral blood (genomics). Dysbiotic samples were characterized by an enrichment of facultative anaerobes with the increase of time, such as AIEC, and a depletion of beneficial species (*Roseburia hominis, Ruminococcus torques* and *Ruminococcus gnavus)*. Particularly, it underscored the decreased stability of microbial composition and immune responses (e.g. cytokines and interleukins) over the course of only weeks in individuals with IBD. Additionally, the PROTECT cohort investigated the role of the gut microbiome in the disease course of 405 pediatric, treatment-naive patients with UC.^127^ Patients were tracked for 1 year (0, 4, 12, and 52 weeks after treatment initiation), and microbial data was analyzed from both fecal and rectal samples. Disease progression biomarkers including anti-*Saccharomyces cerevisiae* antibody (ASCA) immunoglobulin A(IgA), ASCA immunoglobulin G(ASCA-IgG), anti-outer membrane porin C (anti-OmpC), and antineutrophil cytoplasmic antibodies (ANCA) were dynamically associated with the change of *Lachnospiraceae* and *Ruminococcaceae* abundance.^127^ In summary, multiple timepoints tracking on gut microbiota mirrored shifts in host molecular metabolism. However, whether these observations underlie the initiation of disease or rather represent a consequence of that should be sought after with the generation of additional human and experimental evidence.

## Multi-omics integrating methodologies

The increasing availability of multi-omics datasets, generated through high-throughput technologies and large-scale cohorts, has necessitated the development of effective computational methods. Here, we present some key examples of bioinformatic approaches that can be leveraged with the goal of integrating multi-omics data.

### Host-microbiota interaction database

There have been plenty of databases (Table 3) compiling experimentally validated RNA–RNA, protein–protein and metabolite-protein interactions between the human host and diverse microbes. For example, ViRBase, a comprehensive viral genome database, provides detailed information on the sequences of viruses. It was designed to store, curate, and share viral genome sequences, as well as associated metadata such as viral taxonomy, hosts, geographic locations, containing 827,105 virus-host non-coding RNA-associated interaction entries with annotations (e.g., RNA annotations, single-nucleotide polymorphism (SNP), and drug-associated information).^132^ The Host-pathogen interactions database (HPIDB) records over 60,000 unique human protein–protein reactions from experimental and computational studies with the goal of identifying potential therapeutic targets.^133^ MetalinksDB, an open-source database of intercellular metabolite-protein regulations, provides biological annotations about pathways, diseases, and tissues.^134^ Causal Oriented Search of Multi-Omics Space (COSMOS) contains causal paths between metabolites, metabolic enzymes, and host transcription factors using at least two omics modalities from metabolomics, phosphoproteomics, and transcriptomics data.^135^ Collectively, combining these well-established resources could help simulate the interactive biomolecular networks within the human body, providing valuable knowledge for cohort studies and downstream experimental studies. Meanwhile, a major challenge with using multiple databases is the frequent updates and changes to data formats, which can lead to inconsistencies and difficulties in maintaining the continuity of research. Thus, it is essential to implement standardized procedures for database version control and comprehensive documentation. Furthermore, integrating multiple stable and well-maintained databases and cross-validating results across different versions can help mitigate the issues caused by frequent updates, ensuring consistency and reliability in data-driven studies.

**Table 3.** Overview of host-microbiota interaction database.

Database	Description	Number of records	Link
ViRBase	Host ncRNA-virus interactions	827,105 virus-host non-coding RNA-associated interaction entries	http://www.virbase.org/.
VirusMentha	Host-virus and virus-virus protein-protein interactions	15,967 protein-protein interactions between 5,828 proteins	https://virusmentha.uniroma2.it/.
HPIDB	Host-pathogen interactions	69,787 protein-protein interactions (66 host and 668 pathogen species)	https://hpidb.igbb.msstate.edu/.
PHI-base	Host-pathogen interactions	27,974 protein-protein interactions (220 host and 275 pathogen species)	http://www.phi-base.org/.
MetalinksDB	Metabolite-protein interactions	10,165 metabolite-receptor interactions	https://metalinks.omnipathdb.org/.
COSMOS	Host-microbe metabolite-protein interactions	/	/

### Multi-dimensional reduction methods

Multi-dimensional reduction methods have been employed to address the bias of data combination that arise from inherent properties of the data (e.g., scale or sequencing depth) and biological factors (e.g., individuals, species, *etc*.) between different types of multi-omics. For example, Multi-Omics Factor Analysis (MOFA) is a Bayesian unsupervised integration method for multi-omics data in terms of latent factors. These factors capture major sources of variation across different data layers, thereby identifying shared omics features mostly contributing to traits of interest.^136^ Another example includes multiple co-inertia analysis (MCIA) which uses a covariance optimization criterion to transform diverse sets of features (such as genes, proteins, miRNAs) into the same scale and simultaneously projects multiple data sets into the same dimensional space.^137^ Nevertheless, the interpretation of results can be challenging due to the complexity of aligning different data types, and issues like data quality and missing values can hinder accuracy. To overcome these challenges, it is crucial to combine multiple approaches, such as statistical modeling and machine learning, to validate and refine the findings, ensuring robustness and reliability.

### Quantitative trait locus (QTL) analyses

Quantitative trait locus (QTL) analyses can be utilized to integrate genetic data with various other types of multi-omics, such as gene expression QTL (eQTL), protein QTL (pQTL) and microbiome QTL (mbQTL). These analyses are used to identify genomic loci that can explain molecular variation. For example, one study integrated genotype and gene expression data to identify 190 inflammation-dependent *cis*-eQTLs in patients with IBD, highlighting the genetics-inflammation co-affecting intestinal transcription.^138^ Another study integrated genotype, proteomics and microbial data to assess genomic-protein associations exposed to certain gut taxa. For example, a *FUT2* pQTL was associated with reduced abundances of butyrate-producing bacteria, indicating IBD genetic susceptibility could be attenuated or exaggerated by gut microbiota.^139^ Additionally, another study combined host genomics and microbial metagenomics data and identified 12 immune-related mbQTLs, revealing both common and rare genetic variants affecting the immune system that could also be key factors in shaping the gut microbiota in the context of IBD.^129^ However, a significant limitation of current QTL data is that most studies rely on bulk tissue samples, which lack the resolution needed to capture genetic regulatory variation in specific cell types and states. Furthermore, QTL mapping is primarily designed to identify the effects of common genetic variants. Thus, the ability to detect associations for variants with low frequencies is limited, posing a challenge for uncovering the full complexity of genetic regulation. Given these limitations, it is essential to integrate QTL mapping with other techniques, such as functional genomics, to enhance the precision and reliability of the identified loci.

### Artificial intelligence (AI)

AI-based omics integration represents a transformative advance that has the potential to significantly enhance our ability to analyze and interpret complex biological data.

On the one hand, autonomous machine- and deep learning capabilities allow AI to train itself to recognize specific patterns using multidimensional data. This ability enables AI to make predictions of response to therapy or disease progression based on historical data. For example, based on a random forest (RF) classifier, one study constructed a general noninvasive microbiome-based diagnosis model for active UC and CD from eight different populations.^140^ Meanwhile, clinical trials have successfully used AI in IBD endoscopy, including computer-aided detection (e.g., polyp detection), computer-aided diagnosis (e.g., polyp classification),^141^ and improvement (e.g., scoring bowel preparation).^142^ For example, deep convolutional neural network (CNN) has been used to train a prediction model from a set of 5,476 images, automatically providing the endoscopist with accurate evaluation bowel preparation.^143^ AI can also be used to quantify the percentage of visualized colonic surface area, report on the clarity of the endoscopic images,^144^ and identify artifacts and restore distorted visual data.^145^

On the other hand, rapid advances in machine learning have made it possible for many tasks that were once time-consuming and highly uncertain to be now done efficiently on computers.^146^ For instance, the AlphaFold 3 model with a substantially diffusion-based architecture was capable of predicting the joint structure of complexes including proteins, nucleic acids, small molecules, ions and modified residues. With the help of AlphaFold 3, researchers can avoid tedious experimental steps, saving a lot of time and resources.^147^ At the same time, RoseTTAFold incorporated deep learning algorithms that allow researchers to predict the three-dimensional structure of proteins with higher accuracy, which not only improved research efficiency but also improved the understanding of protein–protein interactions.^148^ In general, AI has been widely used in clinical and experimental research thanks to its excellent automatic learning and computing capabilities, which would greatly contributed to improve diagnostic efficiency and undertake a large number of repetitive tasks.

Meanwhile, AI models are also prone to overfitting, which can hinder their application to complex diseases like IBD. To ensure the stability and robustness of the findings, multi-center validation is essential. This approach helps to confirm the generalizability of the models and reduces the risk of biases associated with single-center studies.

## Challenges and perspectives

Although significant advances have been made over recent years, there are specific challenges remaining in current interactome studies.

First, the complex intestinal structures contain numerous “interactome niches”. CD is a disease characterized by transmural inflammation and affecting segmental sites along the entire gastrointestinal tract, while disease lesions in UC are typically limited to the colon and superficial mucosal layers. One study collected tissue samples across the whole intestinal layer and described the presence of distinct microbiota-protein links from multiple locations.^149^ Moreover, a recent study has expanded our recognition on human gut spatial heterogeneity. For example, it first describeed the serrated and branched villi enriched in the small intestine, by constructing a comprehensive spatial expression atlas using healthy tissues.^150^ Another study demonstrated that the spatial landscape of the intestine was robust to the influence of the microbiota and could adapt in a spatially confined manner, which crosstalk between immunity and structural cell homeostasis.^151^ However, whether these physiological features participated in host-microbiota cross-talk under IBD is still unknown and newly developed spatial host – microbiota sequencing may hold the key to decoding the pathogenesis.^152^

Second, uncovering previously unknown information has been relying on re-use and deep mining of existing multi-omics data. For example, microbiome-wide association studies (MWAS) analysis revealed that 1,358 SNPs of bacteria were correlated with host BMI. Different from the previous emphasis on the influence of host variations on microbiota, MWAS analysis revealed the correlation between microbial genetic variations and host phenotype from a new perspective, emphasizing the importance of further research on microbial genetic variations.^153^ In addition, the study of transcriptomics was not limited to the effect of RNA abundance on the host. Another study has constructed a comprehensive map of the human spliceosome regulatory network by re-analyzing bulk transcriptome data, revealing extensive and complex cross-regulatory mechanisms.^154^ Researchers have identified the effects of alternative splicing from the same gene on disease progression, which were often ignored at the level of transcriptional abundance in past studies. Much of the focus on proteomics has been on the discovery of PPI (protein–protein interactions) networks based on co-abundance or co-occurrence. A recent study developed RoseTTAFold2-Lite to identify novel bacterial PPI through predicting the structures of protein complexes.^155^ Low consistency across platforms has been identified due to technical heterogeneity, which limits the development of potentially useful biomarkers in IBD. The manufacturers and companies developing these technologies should put more effort in making their platform more accessible and comparable to other technologies.

Third, although current cohorts with multi-omics data have provided unique opportunities to solve clinical questions, there are still bottlenecks. Due to the limitations of single-center studies or small sample sizes, future cohort studies should prioritize the study of diverse populations.^156^ Human gut microbiomes across different populations contain many common core microbial species. However, within a species, certain strains show notable differences based on the population. This population-specific variation could be the result of a shared evolutionary relationship, referred to as codiversification, between humans and their microbiota. Research examining paired gut metagenomes and human genomes from individuals in Europe, Asia, and Africa has shown that humans and their gut microbes share a parallel evolutionary history.^157^ Another study collected metagenomic data from 30 provinces in mainland China and observed region-specific coexisting MAGs (metagenome-assembled genomes) as well as MAGs with probiotic and cardiometabolic functionalities.^158^ These findings highlight the importance of understanding population-specific microbial strains in microbiome-mediated disease phenotypes. Therefore, it is crucial for future IBD research to incorporate diverse, geographically distinct populations. Additionally, most patients included in current cohorts have already undergone drug or surgical treatments, which may have altered the original molecular manifestations of the disease. For this reason, establishing inception cohorts of treatment-naive patients with IBD is also an important research direction.

Fourth, there have been massive efforts toward making AI and big data commonplace in clinical and biomedical research. As reported in many studies, FMT was a promising therapeutic approach for treating CD.^13^ One study showed that it was possible to explore the donor-bacterial strain engraftment in recipients of the FMT by assessing the bacterial SNVs.^159^ Also, the response to anti-integrin treatment^97^ and anti-TNF therapy^160^ can be predicted based on a combination of the gut microbiome and other clinical factors.^161^ To fully leverage the potential of predictive models in IBD, it is crucial to determine which biomarker characteristics should take precedence. The primary challenge in identifying novel biomarker signatures with clinical value is the complexity and difficulty of translating them into clinical practice.^162,163^ Therefore, prioritizing the development of biomarkers that are easy to use, cost-effective, and reproducible is crucial. Achieving this will require repeated external validation to assess their reproducibility and generalizability.^164^ Meanwhile, many clinicians remained cautious about predictive approaches, primarily because some of AI methods function as “black boxes,” offering predictions without linking them to underlying mechanisms. They also failed to provide functional explanations for the discovered associations, correlations, and recommended decisions. Therefore, experimental and clinical validations are necessary before implementation in clinical settings. In addition, exposome should also be incorporated in big data integration. External environmental factors, such as diet, may also influence clinical outcomes. For example, a recently published multi-omics-based study within an inception cohort of patients with CD demonstrated that adherence to the Mediterranean diet is associated with beneficial clinical outcomes and lower systemic inflammation. The authors showed that this association may be driven by lower dysbiosis and levels of primary bile acids, concurrent with a more favorable microbial and metabolite composition.^165^ In addition, it is important to note that other recent studies have shown that similar dietary interventions may yield different outcomes in the progression and treatment of intestinal inflammation in patients with IBD.^166,167^ Since only little evidence is currently available to support evidence-based dietary recommendations that could lead to greater remission rates in patients with IBD, more studies are needed that work toward developing more advanced dietary strategies. In this regard, the introduction of “personalized dietary therapy” - tailoring dietary therapy for patients with IBD – could be envisioned, potentially realized through gut microbiota-based stratification.^168^

In conclusion, vast amounts of multi-omics data will be continuously generated in the future and will prove helpful to the understanding of the host-microbiota interactome in IBD. Standardized data management, multi-center collaboration, the generation of in-depth clinical metadata, and the use of advanced computational methods are urgently needed to overcome the ceiling effects of clinical translation to improve patient outcomes.

## Supplementary Material

Supplemental Material

## Data Availability

Data sharing is not applicable to this article as no new data were created or analyzed in this study.
