# Supplementary material for: Integrating multi-omics data to reveal the host-microbiota interactome in inflammatory bowel disease
Source: Gut Microbes. 2025 Mar 10;17(1):2476570. doi: 10.1080/19490976.2025.2476570 (PMC11901428; doi:10.1080/19490976.2025.2476570)
Supplement: Supplemental Material [file KGMI_A_2476570_SM2491.docx]

**Table 1. Overview of key multi-omics technologies including a brief description and examples of platforms commonly used for each technology.**

| **Omics** | **Description** | **Example of technological platforms used** |
| --- | --- | --- |
| Metagenomics | Culture-independent analysis of genomic sequences from all microbes in a sample; reveals information on the taxonomic and functional profiles of microbial communities. | Ion Torrent Semiconductor Sequencing,  Illumina Sequencing,  Sequencing by Oligonucleotide Ligation and Detection,  DNA Nanoball Sequencing,  PacBio Single Molecule Real-Time (SMRT) Sequencing,  Nanopore DNA Sequencing |
| Metatranscriptomics | Culture-independent analysis of microbial community transcriptomes; discerns active microbes and their functional expressions under specific conditions. |  |
| Proteomics | Systematic analysis of the proteome; elucidates protein interactions, functions, and structures to provide deeper insights into cellular activities and organismal function beyond genomics. | Mass Spectrometry,  Antibody Capture-based Techniques,  X-ray Crystallography,  Nuclear Magnetic Resonance Spectroscopy,  Liquid Chromatography |
| Metabolomics | Systematic quantification and characterization of small-molecule metabolites in biological samples, reflecting the metabolic state and functional outputs of cellular processes. | Mass Spectrometry,  Nuclear Magnetic Resonance Spectroscopy,  Liquid Chromatography,  Gas Chromatography,  Capillary Electrophoresis |
| Spatial transcriptomics | Omics technique building upon in situ hybridization; captures the spatial context of transcriptional activity within intact tissues and quantifies all mRNA in cells to provide a comprehensive view of cellular processes and their spatial organization. | Multiplexed fluorescence in situ hybridization (M-FISH),  Single-cell RNA Sequencing |
| Spatial proteomics | High-resolution analytical approach that integrates proteomic profiling with spatial localization, elucidating protein distribution, dynamics, and interactions within cellular and tissue contexts. | Cyclic Immunofluorescence (CycIF),  Co-detection by Indexing (CODEX),  Iterative Bleaching Extends Multiplexity (IBEX),  Imaging Mass Cytometry (IMC),  Multiplexed Ion Beam Imaging (MIBI),  Antibody-Based Imaging,  Fluorescent Protein-Based Imaging |

**Table 2. Characteristics of population-level microbiome cohort studies in IBD**

| **Cohort name** | **Numer of participants** | **Data generated** | **Study features** | **Ref** |
| --- | --- | --- | --- | --- |
| RISK(USA) | n=1,276 | Genomics,  Transcriptomics,  Microbiomics;  Blood,  Biopsies,  Faecal samples | Longitudinal, CD only, pediatric inception cohort, multi-omic analysis | PMID: 38490347 PMID: 38289995 |
| PRISM(USA) | n=161 | 16S rRNA sequencing, Metabolomics, Microbiomics; Faecal samples | Longitudinal, | PMID: 30531976 PMID: 29404425 |
| Dutch IBD biobank(Netherlands) | n=3,388 | Genomics,  Transcriptomics,  Microbiomics; Serum,  Faecal,  Mucosal biopsies sample | Cross-sectional, multi-omic analysis | PMID: 29122790 |
| The Swiss Inflammatory Bowel Disease Cohort Study (SIBDCS,Switzerland) | n=3,577 | 16S rRNA sequencing,  Genomics,  Microbiomics; Blood,  Faecal,  Biopsies samples | Epidemiology, multi-omic analysis | PMID: 30689927 |
| IBD BioResource (UK) | n=36,126 | Genomics;  Serum plasma samples | Longitudinal | PMID: 31270165 |
| 1000IBD(Netherlands) | n=1,215 | 16S rRNA sequencing,  Genomics,  Stool,  Biopsies samples | Cross-sectional,  multi-omic analysis | PMID: 30621600 |
| PANTHER（Belgium） | \ | Genomics;  Stool,  Serum,  Mucosal biopsies sample | Longitudinal,  Multi-center,  follow-up | PMID: 31681784 |
| Human Microbiome Projec 2 (HMP2,USA） | n=132 | Microbiomics;  Stool,  Blood,  Biopsy samples | Longitudinal,  Multi-omic analysis | PMID: 31142855 PMID: 38563656 |
| The Crohn’s and Colitis Canada Genetic Environmental Microbial project(GEM project,Canada) | n=3,483 | 16S rRNA sequencing, Metabolomics, Microbiomics; Faecal samples | Family-based,  prospective cohort study of healthy first-degree relatives(FDRs) of individuals with CD | PMID: 37263307 |
| TWIN-IBD (Netherlands) | \ | Blood,  urine,  feces,  Oropharyngeal swabs,  Rectal, colonic or ileal biopsies samples | Family-based,  ongoing,  prospective,  Longitudinal, follow-up, twins only ≥16 years of age | PMID: 33476671 |
| The Inflammatory Bowel Disease Family Cohort (IBD-FC, Germany) | n=1,715 | Fecal,  Blood samples | Family-based,  Prospective, follow-up | PMID:33310084 |
| Predicting Response to Standardized Colitis Therapy (PROTECT, USA and Canada) | n=431 | 16S rRNA sequencing, Metabolomics, Microbiomics; Faecal,  Biopsies samples | Treatment-naive paediatric UC patients | PMID:30308161 |

Note: IBD, inflammatory bowel disease; CD, Crohn's disease; UC, ulcerative colitis;

**Table 3. Overview of host-microbiota interaction database**

| **Database** | **Description** | **Number of records** | **Link** |
| --- | --- | --- | --- |
| ViRBase | Host ncRNA-virus interactions | 827,105 virus-host non-coding RNA-associated interaction entries | http://www.virbase.org/ |
| VirusMentha | Host-virus and virus-virus protein-protein interactions | 15,967 protein-protein interactions between 5,828 proteins | https://virusmentha.uniroma2.it/ |
| HPIDB | Host-pathogen interactions | 69,787 protein-protein interactions (66 host and 668 pathogen species) | https://hpidb.igbb.msstate.edu/ |
| PHI-base | Host-pathogen interactions | 27,974 protein-protein interactions (220 host and 275 pathogen species) | http://www.phi-base.org/ |
| MetalinksDB | Metabolite-protein interactions | 10,165 metabolite-receptor interactions | https://metalinks.omnipathdb.org/ |
| COSMOS | Host-microbe metabolite-protein interactions | / | / |
